# Ultrastructural Changes in Final Instar Larvae of *Papilio polytes* (Lepidoptera: Papilionidae) Lead to Differences in Epidermal Spreading of Water and Adjuvants

**DOI:** 10.3390/biomimetics10040251

**Published:** 2025-04-19

**Authors:** Zhengyu Lu, Xue Wu, Tingting Zhang, Chufei Tang

**Affiliations:** 1 College of Plant Protection, Shandong Agricultural University, Taian 271018, China; 2023120132@sdau.edu.cn; 2 Institute of Leisure Agriculture, Jiangsu Academy of Agricultural Sciences, Nanjing 210014, China; wuxue@njfu.edu.cn; 3 College of Forestry and Grassland, Nanjing Forestry University, Nanjing 210037, China

**Keywords:** *Papilio polytes*, larvae, epidermal ultrastructure, wetting, pesticide adjuvants

## Abstract

*Papilio polytes* is a cosmopolitan Lepidoptera species of controversial use and management. It remained unclear how its epidermal ultrastructure changes during development and how this affects its wetting properties in relation to water and pesticide adjuvants. In this study, the epidermis of *P. polytes* was systematically examined at the important feeding stage (from 3rd to 5th instar). Its ultrastructure was quantitatively observed by scanning electron microscopy. Its wetting properties towards the three main types of adjuvants and water were evaluated by contact angle. The chemical functional group differences between different instars and different adjuvant treatments were analyzed by mid-infrared spectroscopy. The correlation between the ultrastructural deformation and variations in wetting properties was verified by simulation tests. It was found that the complication of the epidermal structure was the leading factor for the significant increase in hydrophobicity during development. Cationic adjuvants had the best infiltrating effect on complex epidermal structures and organosilicon adjuvants had the best infiltrating effect on simple epidermal structures. The results provide data for biomimetic design for different wetting properties and suggest the feasibility and advantages of selecting pesticide adjuvants based on developmental changes in the structural characteristics of the insect epidermis.

## 1. Introduction

Butterflies are popular not only for their attractive appearance but also for their ecological importance as pollinators and environmental indicators [1]. However, the larvae of some species are considered agricultural pests due to their feeding habits, being oligophagous or monophagous on certain crops and orchards, and can sometimes cause significant losses to agricultural produce [2]. *Papilio polytes* (Lepidoptera: Papilionidae), the common Mormon, is a well-known cosmopolitan species that has been the subject of much debate on this issue. In addition to their aesthetic appeal and pollination function as swallowtails, these insects are of great research value due to their distinctive Batesian mimicry, which involves intraspecific polymorphism [3]. However, their larvae are also considered pests of Rutaceae, as they feed on the leaves of this plant family [4]. As the population grows, this can lead to a decline in fruit production and threaten the survival of the plants. Understanding their survival characteristics to agricultural activities at different stages of development is essential to formulate targeted measures and thus strike a balance between their management and conservation.

The epidermis of Lepidoptera larvae, including *P. polytes*, despite possessing a thickness of only 1–2 µm, functions as a pivotal barrier between its tissues and the external environment [5]. Its capacity to restrict water movement is contingent not solely on the chemical composition, which in addition to the main framework of chitin and protein complexes, containing lipids (including the cuticle hydrocarbons [CHCs], waxes, phenolic compounds), and inorganic salts; but also on a distinctive ultrastructural configuration that enhances the barrier function by substantially increasing the surface area to volume ratio [6]. Studies have shown that the resistance of the epidermis to wetting can be overcome by adding adjuvants to promote the distribution of contact pesticides [7]. For those popular in controlling the population of Lepidoptera larvae, organosilicon adjuvants enhance the spreading and adhesion of the liquid by reducing surface tension [8], cationic salts enhance the uniformity of the liquid distribution by regulating the ionic environment [9], and lipid adjuvants promote the penetration of pesticides by mimicking the lipid structure of the epidermis [10].

The functional morphology and composition of the epidermis of Lepidoptera larvae undergo dynamic changes during larval development, and the differences are especially obvious before and at the final instar stage [11,12,13]. The process of epidermal tanning significantly enhances the epidermis’ resistance to external substances, a phenomenon that is not as evident at other instars [6,14]. This observation indicates that the epidermis may be subject to synergistic regulation of barrier properties by constituents (e.g., tanning-related molecules) and the ultrastructure during development [15,16]. This dynamic regulation is likely to influence the wettability characteristics of Lepidoptera epidermis, and, therefore, be involved in shaping the difference of efficacy of fixed pesticide and adjuvant combinations in different instars [17]. However, the developmental dynamics of the epidermis of Lepidoptera larvae (e.g., ultrastructural and compositional differences of different developmental stages) and their specific effects on wettability are not yet fully understood, and the relationship between epidermal characteristics and developmental stages needs to be further investigated in order to guide the precise design of adjuvants and optimization of pesticide application strategies. Priority should be given to the epidermis surrounding the spiracle, which is the gas exchange channel on the insect body surface that connects to the tracheal system and is therefore an important route for contact insecticide entry into the body, and the wetting properties of the surrounding epidermis can significantly influence the amount of insecticide to which the spiracle is exposed.

In the study of surface wetting effects, contact angle (θ) is a key parameter to characterize the degree of wetting [18]. Based on the classical wetting theory, Young’s Equation describes the equilibrium contact angle of a droplet on an ideal smooth surface, and its value is determined by the interfacial energy of solid-liquid-gas [19]. According to the size of the contact angle, the wetting behavior can be classified into three categories: (i) θ < 90°: the liquid spreads out on the surface, which is hydrophilic; (ii) 90° < θ < 150°: the liquid tends to form droplets, which is hydrophobic; (iii) θ > 150°: the liquid hardly spreads out, and forms near-spherical droplets, which is superhydrophobic. Young’s theory provides a fundamental framework for the study of wetting phenomena and is widely used in physics, chemistry, biology, and materials science [20]. However, real surfaces usually have non-ideal factors such as roughness and chemical heterogeneity, which lead to contact angle hysteresis (CAH), i.e., the actual contact angle deviates from the predictions of Young’s theory [21]. For this reason, the Cassie–Wenzel theory introduces the effect of surface roughness, which corrects the solid–liquid effective contact area and thus describes the wetting behavior more accurately [22]. In recent years, studies have further penetrated into the molecular scale and revealed the microscopic nature of the wetting phenomena by analyzing the asymmetry of the liquid–air density distribution, the fluid–solid van der Waals interactions, the liquid–solid and solid–gas interfacial tensions, and the molecular rearrangement at the interface [20,21,23]. These theories not only explain the wetting differences of different liquid-surface combinations but also provide a theoretical basis for predicting the wetting behavior of complex surfaces [24,25]. In order to simulate the wetting effect, various computational tools have been developed, such as LAMMPS, which is applicable to the simulation of nanoscale wetting behavior with molecular dynamics as the core [26], ANSYS Fluent, and COMSOL Multiphysics, which are applicable to the analysis of macroscopic droplet dynamics with computational fluid dynamics as the core [27,28]. Each of these tools has its own advantages in terms of computational accuracy, applicability and resource requirements, and can provide multi-scale analytical support for specific problems (including the wetting behavior of additives in insect epidermis), thus laying a solid foundation for the study of wetting mechanism.

In this study, we focus on the epidermis of the tergum surrounding the spiracle of 3rd to 5th instar larvae of *P. polytes*, the stage at which food intake is relatively high and can have adverse effects on host plants. Its ultrastructure, composition, and wetting properties in relation to water and three adjuvant types (organosilicon, cationic, and lipids) were investigated and compared between different instars. The relationship between cuticular ultrastructure and wetting properties was verified by analyzing the difference in cuticular ultrastructure and components before and after adjuvant application and the associated virtual simulation. The results will provide a reference for the field management of *P. polytes* under different conditions and at different instars, and provide insights into understanding the changes and related functions of cuticular ultrastructure of lepidopteran larvae during development.

## 2. Materials and Methods

### 2.1. Materials

#### 2.1.1. Insects

The specimens were fed a strict diet of leaves from *Citrus limon* and were bred in a controlled environment with strict regulation of light, temperature, and moisture (25.0 ± 1.0 °C, relative humidity 75.0 ± 5.0%, light:dark = 12:12). The collection of specimens occurred within 2 h following ecdysis in order to minimize the potential impact of developmental changes. Subsequent to collection, all specimens were stored in 75% alcohol at a temperature of 4 °C.

#### 2.1.2. Adjuvants

In this study, three adjuvants were utilized: organosilicon adjuvants, represented by two commercial adjuvants that function with alkoxylated trisiloxane: 0.03% Silwet 618 and 0.03% Silwet 408 (100%, Momentive Performance Materials Inc., Nantong, the Economic & Technological Development Zone); cationic adjuvants, represented by 1.0 g/L tetradecyl benzyl dimethyl ammonium chloride (1227, 44%, Shandong Usolf Chemical Technology Co., Ltd., Linyi, Lanshan) and 0.9 g/L benzalkonium chloride (1427, 99%, Shanghai Maclin Biochemical Technology Co., Ltd., Shanghai, Fengxian); and lipidic adjuvants, represented by 1.0% methylated seed oil (Maifei, 100%, Beijing Guangyuan Yinong Chemical Co., Ltd., Beijing, Haidian), 0.1% octodecyl glucoside (APG0810, 50%, Shandong Usolf Chemical Technology Co., Ltd., Linyi, Lanshan), and 97% mineral oil (97%, Shanghai Hulian Bio-Pharmaceutical (Xiayi) Co., Ltd., Shangqiu, Xiayi). The adjuvants were dissolved in water at a temperature of 20.0 ± 1.0 °C using a vortex oscillator and stored at a temperature of 4 °C. Due to the differences in the physical form of different adjuvants (e.g., liquid, solid, etc.), the above dilution methods (including dilution by mass fraction, volume ratio, or mass/volume ratio) and their specific proportionality parameters are referred to the actual application programs recommended by the respective product specifications.

#### 2.1.3. Sampling

**Figure 1:**
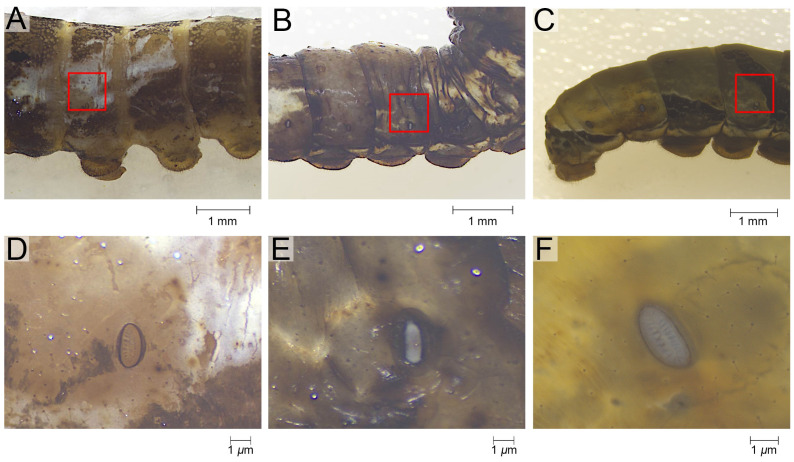
The sampling of the epidermis of *P. polytes*. (**A**–**C**) The sampling range annotated with the red rectangle. (**D**–**F**) The morphology of the specimen as microscoped. (**A**,**D**) the 3rd instar, (**B**,**E**) the 4th instar, (**C**,**F**) the 5th instar.

The epidermis of the tergum 3rd to 5th instar larvae was studied. Sampling was performed vertically, with the ventral edge of the spiracle as the boundary, extending 0.4 cm dorsally, and horizontally, with the center of the spiracle extending 0.2 cm anteriorly and posteriorly (Figure 1A–C). This method made it easy to maintain the consistency of the sampling area across different instars, and the sampling area was able to meet the material requirements of the series of experiments performed on the same samples. A pre-test was performed to check the similarity of ultrastructure, wettability, and composition of epidermis from this region of different body segments. Sampling was therefore restricted to the 3rd to 5th abdominal segments, which are relatively large. Prior to the experiments, the epidermis was peeled off under a stereoscope (Zeiss SteREO Discovery V.8, Gottingen, Germany).

#### 2.1.4. Treatments

The samples were soaked in hexyl hydride (analytically pure) for 24 h to observe the ultrastructure of chitin, i.e., the epidermal structure without the CHCs. For the samples resolving the influence of adjuvants, these were soaked in the representatives of the three types of adjuvants for 2 h after being dried, and then rinsed, fixed, dehydrated, and dried as above. For the study, 1% methylated seed oil, 0.9 g/L benzalkonium chloride, and 0.03% Silwet 618 were chosen as the representative adjuvants as the wetting properties of the same type adjuvants are similar (see Section 3.2). Samples with and without the above treatments were all then rinsed using 0.1 mol/L phosphate buffer (pH = 7.2), followed by fixation with 2.5% glutaraldehyde solution for 48 h. The epidermis was subsequently dehydrated using gradients of ethanol solutions (75%, 80%, 90%, and 95%), with a 20 min soak under each gradient, and finally, dried naturally.

### 2.2. Microscopy

For the observation of the surface, the samples were directly affixed to insulating tape. For the observation of the section, the samples were additionally secured with acrylic resin in order to maintain an upright position. Prior to observation, all dried samples were sputtered with gold. All observations were conducted under a scanning electron microscope (Zeiss EVO-LS10, Gottingen, Germany) with an acceleration voltage range of 5–15 kV. Five replicates were used for each sample.

### 2.3. Morphometric Measurements

All measurements were obtained from the photographs using FIJI-Image J (64-bit Java 1.8.0_172) [29]. The program can count the pixels occupied by a region or line and convert them to actual size using plotting scales. The line and angle tools were used to quantitively describe the size and shape of the pillar and scale-like arrays as well as the distance between them. Each morphological parameter was measured with 20 replicates.

### 2.4. Wetting Property Measurements

**Figure 2:**
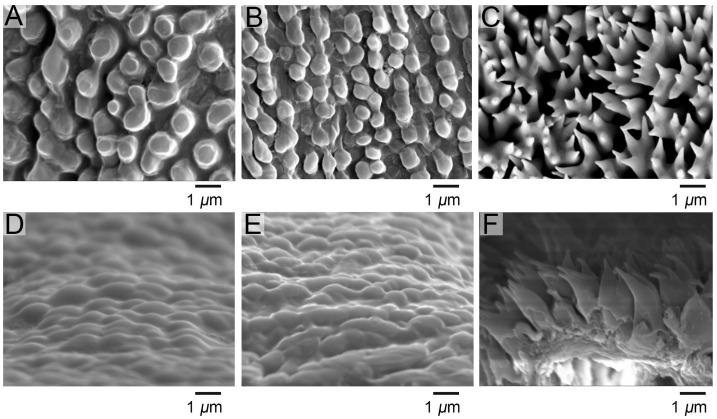
Ultrastructure of the epidermis of *P. polytes*. (**A**–**C**) Surface ultrastructure under scanning electron microscopy; (**D**–**F**) Section ultrastructure under scanning electron microscopy; (**A**,**D**) the 3rd instar, (**B**,**E**) the 4th instar, (**C**,**F**) the 5th instar.

The contact angle of water and the adjuvants on the samples from the 4th and 5th instar larvae of *P. polytes* were measured for the assessment of their wetting characteristics, as the ultrastructural characteristics of the epidermis of 3rd and 4th instar larvae are identical (see Section 3.1, Figure 2). A contact angle meter (KRÜSS DSA100S, Hamburg, Germany) with the bundled software ADVANCE version 1.14 was used. Samples were affixed to slides using double-sided adhesive. The volume of each droplet was 20 μL, and the contact angle was measured after the droplet was stabilized for 10 s in contact with the sample. Six replicates were measured for each result.

### 2.5. Transmission Spectra Measurements

Mid-infrared transmission spectroscopy at wavelengths ranging from 500 to 4000 cm^−1^ was employed to detect the characteristic signals of the epidermal chemical functional groups of 4th and 5th instar larvae, both untreated and treated with the soaking of representative adjuvants, which could indicate the differences of epidermal components. Transmission spectra were measured using a Fourier transform infrared spectrometer (Thermo Fisher Scientific Nicolet iS50, Madison, WI, USA) with three replicates per sample.

### 2.6. Data Analysis

#### 2.6.1. Significance of Difference

The significance of differences in epidermal morphological characteristics and wetting properties between instars and treatments, as well as differences in contact angle to water and different adjuvants at the same age, were investigated. Duncan’s method was used for parameters that were tested to be normally distributed. Rank-sum test was used for parameters that did not conform to normal distribution (Mann–Whitney U-test for testing differences between 2 groups, Kruskal–Wallis test for testing differences between 3 groups and above). The above tests were performed in IBM SPSS Statistics 25.

#### 2.6.2. Chemical Functional Groups Comparison

The mid-infrared transmission spectra were imported into Origin Pro2025 for composition determination. The baselines of the spectra were corrected using the constant mode in the “baseline” tool, and then the “Peak Analyzer” was used to identify the positions and intensities of the absorption peaks and to identify the characteristic chemical signals of the epidermal components based on the molecular vibrational theory.

#### 2.6.3. Simulation of Contact Angles

The COMSOL Multiphysics 6.2 two-phase laminar flow model, which simulates wetting based on Young’s, Wenzel, and Cassie–Baxter equations, was used to simulate the contact angles of water and three representative adjuvants on 4th and 5th instar larval epidermis without hydrostatic pressure compensation. This was carried out to analyze the pressure changes inside the droplets during the contact process and to validate the correlation between the ultrastructure and the wetting properties of the epidermis. The background flow phase field was set to air (using the built-in system parameters). During the simulation process, the mean and standard deviation of the measured contact angles (see Section 3.2, Table 1) were used as the static values and dynamic hysteresis ranges, respectively, and were directly applied as boundary conditions at the solid–liquid–gas junction to calculate the solid-liquid interfacial energy and the solid–gas interfacial energy and encrypted the mesh near the interface to enable adaptive time-stepping until the model converged, and the transient solver was used to capture the droplet dynamic change process.

For the simulated epidermis, its appearance was based on the corresponding geometric characteristics concluded from the morphological observations. Maintaining the same shape, all size-dependent morphology parameters are scaled up by a factor of one thousand. This means that morphology changes from micrometer to nanometer scales are transformed into millimeter-scale morphology changes. This is due to the fact that the droplet diameter in the actual experiment is approximately 3.36 mm (roughly equivalent to 1.24*V^1/3^, V = 20 μL), whereas, in the simulation, the computational limitations stipulate that the dimensions of the surface roughness structure should not exceed 100-fold the droplet diameter. Maintaining the size of the surface morphology and reducing the droplets to the micrometer level would result in additional microscale effects such as wall slippage and evaporation. The dynamics are dominated by the local interaction of surface tension, viscous force, and roughness, which is quite different from the actual situation (dominated by inertial force and gravity). Its material properties were set as the default parameters of chitin in the system, and its surface was set as the only wet wall.

For the liquid properties, in order to avoid additional effects when the ratio of surface structural unit to droplet diameter changes by orders of magnitude, such as the pinning effect, the initial droplet size was scaled up to 10 times the smallest structural unit of change (the width of a furcation of the epidermal scale protuberance of 5th instar larvae, which is about 0.4 μm and isometrically scaled up to 0.4 mm), i.e., 4 mm. The initial drop height was set to 8 mm. In order to maintain the requisite level of kinetic similarity (Reynolds and Weber numbers unchanged), the viscosity and surface tension coefficients were scaled up to 1.30 times (equals to diameter ratio^3/2^, 4/3.36^3/2^ ≈ 1.30) and 1.19 times (equals to diameter ratio, 4/3.36 ≈ 1.19) their original values, respectively. The original values of viscosity and surface tension coefficient are outlined below. The built-in system parameters for water were used, the official product information for the three adjuvants was referred to, and they were set in the order of 1% methylated seed oil, 0.9 g/L benzalkonium chloride, and 0.03% Silwet 618 as follows: surface tension was set to 25, 30, and 22 mN/m; density was set to 0.92, 0.99, and 0.99 g/cm^2^; and kinetic viscosity was set to 75, 1, and 1 mPa·s.

## 3. Results

### 3.1. Comparative Morphology of Epidermal Ultrastructure

The epidermis of both 3rd and 4th instar larvae had continuous and rounded papillae and did not differ significantly in size and shape, but 5th instar larvae had epidermal protuberances in the form of 2–3-lobed scale-like protuberances (Figure 2). The epidermal thickness was 2.01 ± 0.17 μm in the 3rd instar and 2.09 ± 0.47 μm in the 4th instar. In the 3rd instar, the width of the base of the papillae was 0.88 ± 0.05 μm, the height was 0.65 ± 0.01 μm, the spacing between the protuberances was 1.23 ± 0.14 μm, and the angle of the protuberance to the epidermis was 39.44 ± 1.36°. In the 4th instar, the base of the papillae was 0.90 ± 0.06 μm, the height was 0.67 ± 0.03 μm, the spacing was 1.27 ± 0.52 μm, and the angle between the protuberance and the epidermis was 40.05 ± 9.43°. The epidermal thickness of 5th instar was 2.77 ± 1.01 μm, the width of the base of the scale-like protuberances was 1.18 ± 0.01 μm, the width of the tips was 1.43 ± 0.25 μm, the height of the unlobed section at the tips was 1.80 ± 0.25 μm, the height of the lobed section was 0.50 ± 0.07 μm, with spacing of 1.67 ± 0.17 μm, and the angle between the lobes was 49.87 ± 9.54° in those have three lobes, and 60.16 ± 6.35° in those have two lobes. The soaking of adjuvants and hexyl hydride had no significant effect on the epidermal structure (Table A1 and Table A2).

### 3.2. Comparison of Wetting Properties of Epidermal Surface

**Figure 3:**
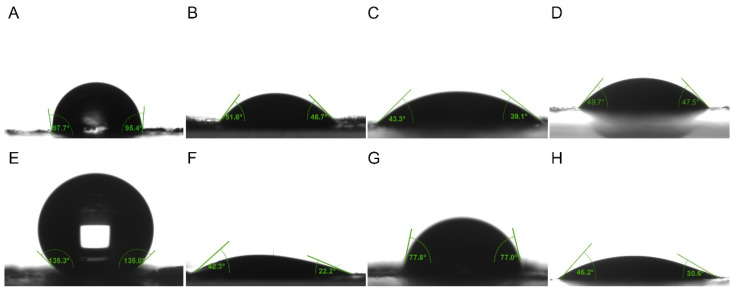
The contact angle of liquids on epidermal surface of 4th and 5th instar larvae of *P. polytes*. (**A**–**D**) Liquids on epidermal surface of 4th instar larvae; (**E**–**H**) Liquids on epidermal surface of 5th instar larvae. (**A**,**E**) Water; (**B**,**F**) 1% methylated seed oil (representative of lipids); (**C**,**G**) 0.9 g/L benzalkonium chloride (representative of cationic adjuvants); (**D**,**H**) 0.03% Silwet 618 (representative of organosilicon adjuvants).

The wetting properties of the epidermis of *P. polytes* larvae exhibited variation between instars and liquid types (Table 1, Figure 3). A pronounced shift in hydrophobicity was observed, progressing from a nearly wetting state (98.03 ± 3.37°) in the 4th instar to a significant level of hydrophobicity in the 5th instar. The application of adjuvants resulted in the spread of liquids across the epidermis, though the effects of the adjuvants varied. The lipidic and organosilicon adjuvants showed stronger infiltration within the epidermis of 4th instar larvae compared to 5th instar larvae, while cationic adjuvants exhibited the opposite trend. The contact angles of most lipidic adjuvants on the epidermis were found to be significantly larger than those of organosilicon and cationic adjuvants. The wettability of organosilicon adjuvants was weaker than cationic adjuvants in 4th instar larvae, but stronger than cationic adjuvants in 5th instar larvae. However, the use of the adjuvants did not significantly change the hydrophobicity of the epidermis itself. The contact angles of water on the epidermis were recorded as 97.96 ± 2.00°, 98.80 ± 1.98°, and 98.36 ± 2.49°, respectively, following treatments with 1.0% methylated seed oil, 0.9 g/L benzalkonium chloride, and 0.03% Silwet 618 on the epidermis of the 4th instar larvae, and 126.87 ± 2.87°, 127.94 ± 5.32° and 126.63 ± 2.57° on the epidermis of the 5th instar larvae, all of which had no significant difference to those without any treatment.

The performance of different adjuvants in terms of infiltration on the epidermis of larvae of different instars also varied (Table 1). Among the lipid adjuvants, 97% mineral oil was weaker than 1.0% methylated seed oil and 0.1% octadecyl glucoside in both 4th and 5th instar larvae. There was no significant difference between 1.0% methylated seed oil and 0.1% octadecyl glucoside in terms of infiltration on 4th instar larvae, but the latter performed significantly better than the former on 5th instar larvae. The latter performed significantly stronger than the former. In a similar manner, the investigation revealed that 0.03% Silwet 408 and 0.03% Silwet 618 exhibited no significant difference in infiltration on 4th instar larvae. However, Silwet 408 showed a significantly stronger performance than Silwet 618 on 5th instar larvae. Furthermore, 0.9 g/L benzalkonium chloride demonstrated weaker performance than 1.0 g/L tetradecyl benzyl dimethyl ammonium chloride on both 4th and 5th instar larvae. Of all the adjuvants, 97% mineral oil exhibited the weakest wettability on both 4th and 5th instar larvae, while 1.0 g/L tetradecyl benzyl dimethyl ammonium chloride demonstrated the most optimal wettability on 4th instar larvae. However, on 5th instar larvae, 0.03% Silwet 408 exhibited the best wettability.

### 3.3. Comparison of Epidermal Chemical Functional Groups

**Figure 4:**
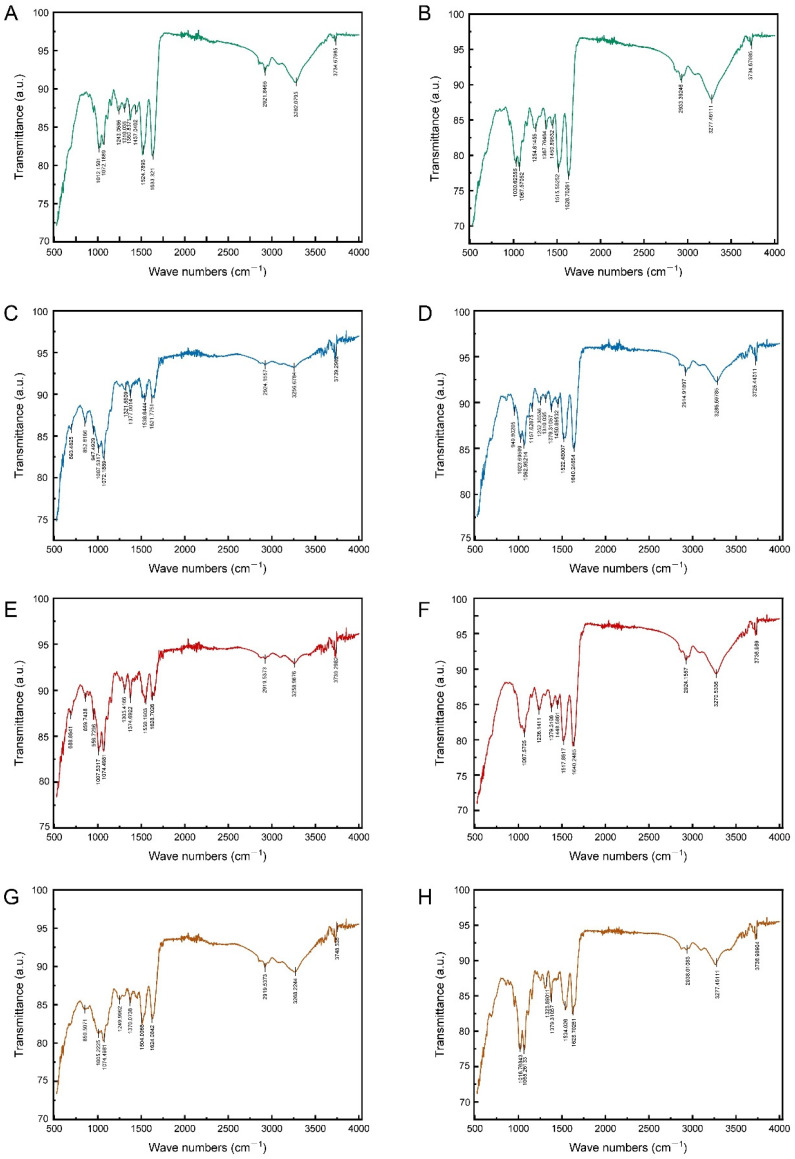
The transmittance spectra of epidermis surface of 4th and 5th instar larvae of *P. polytes*, untreated and treated with different adjuvants. (**A**,**B**) Untreated; (**C**,**D**) treated with 1% methylated seed oil (representative of lipids); (**E**,**F**) treated with 0.9 g/L benzalkonium chloride (representative of cationic adjuvants); (**G**,**H**) treated with 0.03% Silwet 618 (representative of organosilicon adjuvants). (**A**,**C**,**E**,**G**) 4th instar larvae; (**B**,**D**,**F**,**H**) 5th instar larvae.

The mid-infrared spectra of the epidermis of 4th and 5th instar larvae exhibited a remarkable similarity in the characteristic frequency region (1300–4000 cm^−1^), with both spectra displaying signals indicative of functional groups of proteins, chitin, CHCs, and pigments (Figure 4A,B). The N-H stretching vibration (peak at around 3300 cm^−1^), the C=O stretching vibration (peak at around 1600 cm^−1^), and the C-N stretching vibration (peak at around 1300 cm^−1^) were indicative of the chitin–protein complex. Indicators of CHCs were the C-H stretching vibration (peak at about 2900 cm^−1^) and the CH_3_ deformation vibration (peak at about 1360 cm^−1^). The presence of phenols and pigments was indicated by the aromatic C=C stretching vibrations (peaks at 1500–1600 cm^−1^). Chitin was indicated by the C-O stretching vibration (peaks around 1000–1100 cm^−1^) in the epidermis of both instars, too. Nevertheless, some differences were also observed in the fingerprint region (1300–400 cm^−1^). For example, the mid-infrared spectra of the epidermis of 5th instar larvae contained C-H stretching vibration features of aromatic ring substituents (with peaks at about 850 cm^−1^) and C-Cl stretching vibration features of chlorinated hydrocarbons (with peaks at about 690 cm^−1^) (Figure 4B). These findings indicated that there were differences in the epidermal CHCs, phenols, and pigments of different larval instars.

The impact of soaking in different adjuvants on the mid-infrared spectrum of the epidermis was found to be variable. The immersion treatment with 0.03% Silwet 618 solution did not exert a significant effect on the mid-infrared spectral characteristics of the larval epidermis. Neither the position nor the intensity of the absorption peaks was found to be significantly altered. Conversely, lipid and cationic adjuvant treatments produced analogous effects that indicated dissolution and chemical reaction on the mid-infrared spectra of the epidermis of 4th and 5th instar larvae. This treatment effect was consistent with the similarity of the intrinsic spectra of the larval epidermis during the different instars, mainly in the fingerprint region (680–1400 cm^−1^) rather than in the characteristic frequency region (2000–3600 cm^−1^) (Figure 4). The following observations can be made. Firstly, the peaks relating to the chitin–protein complex and chitin showed little difference before and after immersion in the two types of adjuvants. Within the 2000–3600 cm^−1^ eigenfrequency region, the overall spectral profile remained stable. Secondly, significant changes were observed, with characteristic peaks in the 1300–1400 cm^−1^ interval being displaced and altered in intensity. The signals in this region corresponded to the vibrations of the relevant functional groups of the epidermal hydrocarbons (CHCs). Additionally, new signals related to CHCs, phenols, and pigments were detected in the 680–860 cm^−1^ band.

### 3.4. Correlation Between Epidermal Ultrastructure and Wetting Properties

**Figure 5:**
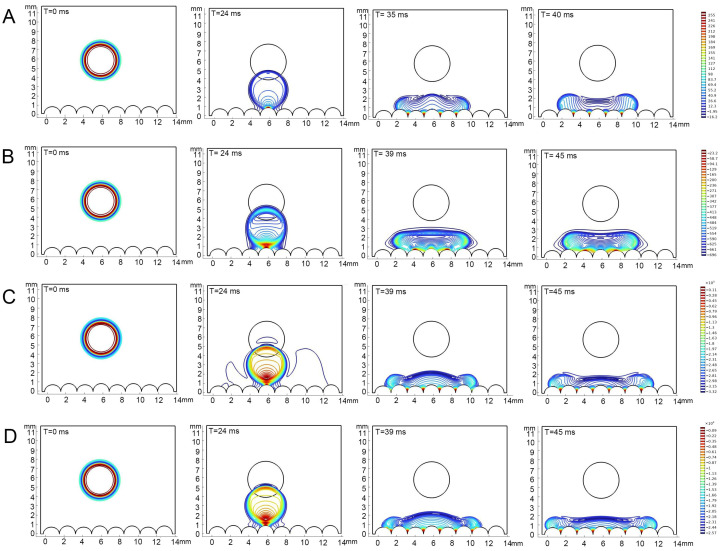
Simulations of dropping process on the epidermis of 4th instar larvae of *P. polytes*. From left to right, the process of drop fall to infiltration is shown, and the fall time is marked in each sub-panel. (**A**) Water; (**B**) 1% methylated seed oil (representative of lipids); (**C**) 0.9 g/L benzalkonium chloride (representative of cationic adjuvants); (**D**) 0.03% Silwet 618 (representative of organosilicon adjuvants). The color indicates the pressure distribution within the droplets, the closer the color is to red, the higher the pressure, the closer the color is to blue, the lower the pressure.

**Figure 6:**
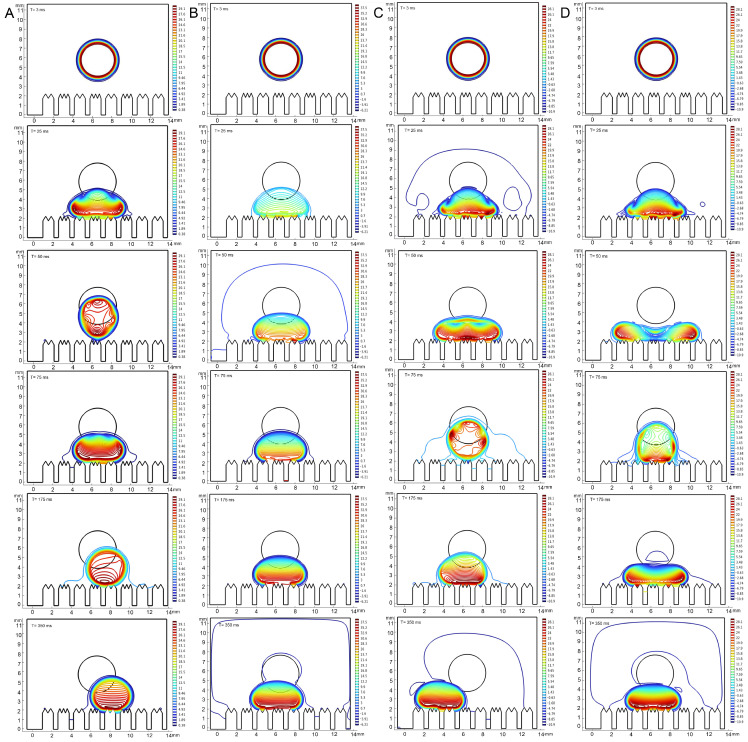
Simulations of dropping process on the epidermis of 5th instar larvae of *P. polytes*. From top to bottom, the process of drop fall to infiltration is shown, and the fall time is marked in each sub-panel. (**A**) Water; (**B**) 1% methylated seed oil (representative of lipids); (**C**) 0.9 g/L benzalkonium chloride (representative of cationic adjuvants); (**D**) 0.03% Silwet 618 (representative of organosilicon adjuvants). The color indicates the pressure distribution of the droplets, the closer the color is to red, the higher the pressure, the closer the color is to blue, the lower the pressure.

The contact angles of each liquid type, as obtained from the simulation, exhibited consistency with the actual measurements. It was observed that all types of liquids did not undergo bouncing on the epidermis of 4th instar larvae and diffusion occurred to varying degrees, with the contact angle of water remaining above 90° (Figure 5). On the epidermis of 5th instar larvae, water showed bouncing behavior and did not infiltrate, whereas adjuvants gradually stabilized after the occurrence of the CAH phenomenon and showed infiltration behavior, though the infiltrating level is distinctly lower than that on the epidermis of 4th instar larvae (Figure 6, Supplementary Materials). On both the 4th and 5th instar larval surfaces, the silicone adjuvant exhibited the greatest spread on the body surface compared to the cationic adjuvant, which also spread to a greater extent than the lipid-based adjuvant. Furthermore, the time for each type of liquid to reach a contact steady state on the surface was found to be similar. The droplets contacted the body surface of 4th instar larvae after 24 milliseconds (ms) and 5th instar larvae after 18 ms. The droplet diffusion process was completed within 45 ms and 350 ms, respectively, on the epidermis of the 4th and 5th instar larvae. These findings substantiated the dependability of the droplet dynamics delineated in the simulations.

The pressure within the drops fluctuated during the drop and contact phases, with these fluctuations varying in magnitude among the liquid types and the instars of the epidermis. In general, the range of pressure variations in droplets was significantly greater on the epidermis of 4th instar larvae than on the epidermis of 5th instar larvae. The range of pressure variations within different droplets was, in descending order, as follows: 0.03% Silwet 618, 0.9 g/L benzalkonium chloride, water, and 1% methylated seed oil. Specifically, pressure changes within the water droplet ranged from 16.2 to 255 Pa (4th instar) and 0.38 to 29.1 Pa (5th instar); within the 1% methylated seed oil droplet, changes ranged from −696 to 23.3 Pa (4th instar) and −6.21 to 37.5 Pa (5th instar); 0.9 g/L benzalkonium chloride intra-droplet pressures ranged from −3.32 × 10^–3^ to 0.11 × 10^−3^ Pa (4th instar) and −10.9 to 28.1 Pa (5th instar); and 0.03% Silwet 618 intra-droplet pressure changes ranged from −2.57 × 10^–3^ to 0.09 × 10^−3^ Pa (4th instar) and −10.9 to 28.1 Pa (5th instar). The pressure distribution within a droplet underwent a progressive change as the droplet fell, and then contacted and infiltrated the epidermis. At the initial stage, the pressures were all predominantly at the outer 0.64 μm of the periphery, decreasing from the core toward the periphery. Upon initial contact with both the 4th and 5th epidermis, the degree of pressure inhomogeneity within the droplets increased, with the pressure increasing as the distance from the body surface decreased. The pressure within the droplet underwent a substantial decrease, accompanied by a considerable reduction in its inhomogeneity, once it had reached a state of equilibrium on the epidermis of 4th instar larvae. However, the pressure inhomogeneity within the droplets barely changed during the wetting process on the epidermis of 5th instar larvae. Nevertheless, in terms of absolute values of pressure, there was little difference in the internal pressure distribution of the same droplet when it reached a steady state on the epidermis of different instars. The aforementioned results suggest that droplet properties and alterations in the morphological structure of the epidermis, play a pivotal role in determining wettability.

## 4. Discussion

In this study, we identified a shift in the ultrastructure of the epidermis in the last instar of *P. polytes* larvae, which plays a major role in altering its wetting properties regarding water and adjuvants. This structural complexification resulted in a significant increase in epidermal hydrophobicity. The application of different adjuvants yielded varied outcomes regarding wetting efficacy. Specifically, the complexity of the epidermal structure diminished the wetting effect for lipid and silicone adjuvants, while cationic adjuvants exhibited the opposite effect. The chemical action of adjuvants did not show a substantial impact on the wettability of the epidermis. This study offers the first systematic quantitative data not only on the epidermal ultrastructure of Lepidoptera larvae and their wetting properties against various pesticide adjuvants but also on the complex changes in relevant properties during their developmental course. Furthermore, it suggests the feasibility and advantages of selecting pesticide adjuvants based on the developmental changes in the structural features of the epidermis of pests.

The variation in the difference in the wetting effect of different liquids upon epidermal complexation stems from their different wetting mechanisms. The hydrophobicity of the insect epidermis, with chitin as the main component, is positively correlated with its surface area [17]. Thus, the increase in area due to structural complication leads to an increase in surface energy and, consequently, enhanced epidermal hydrophobicity. The mechanism of action of organosilicon adjuvants, based on low surface tension and molecular chain flexibility, involves penetration into the wax lattice interstices and along the micro protuberances to complete the spreading [8]. This process is unfavorable for spreading across the complex structure of the epidermis. Lipid-based adjuvants are susceptible to phase solubility effects due to their molecular polarity, which is similar to that of long-chain hydrocarbons in the larval epidermis [10]. Consequently, these adjuvants exhibit relatively poor wetting at low surface energy interfaces, such as an epidermis with a relatively simple structure. Cationic adjuvants have been shown to overcome the hydrophobic barrier through electrostatic adsorption of charged groups with free carboxylic acid groups in the epidermis [9]. The molecular conformational flexibility of this process, involving head-group adsorption and tail-chain wiggling, enables relatively good structural adaptation.

The structural changes showed an independent and important role during the process, and it is possible that this may have multiple biological significance and is closely related to the adaptive needs of different developmental stages [6,30]. However, the final instar larvae of Lepidoptera exhibit a surge in feeding and increased mobility, which coincides with the time window in which the hydrophobicity of the *P. polytes* epidermis increased, as observed in the present study [31]. The thickening and reprogramming of the epidermis balances the static hydrophobicity (high contact angle) with dynamic stability (hysteresis). This can enhance physical defenses against external stresses, such as water and microbes, and improve self-cleaning power [6,17]. Additionally, it may be involved in the regulation of other functional properties, such as the compressive strength and adhesive properties of the epidermis to enhance the mobility of senior larvae in complex vegetation environments, and therefore, provides survival advantage in different ecological environments [32,33]. From the perspective of developmental regulatory mechanisms, the dynamic balance between ecdysone (20E) and juvenile hormone (JH) may dominate this process [34,35,36]. It has been shown that a decrease in JH levels triggers the larvae to enter the final instar stage, in which epidermal cells reprogram the epidermal structure by activating the differential expression of the chalcone synthase gene (CHS) and the cytochrome P450 reductase gene (CPR) [37,38].

Still, the developmental transition in epidermal hydrophobicity in the larvae of *P. polytes* is essentially a result of the cooperation of epidermal ultrastructural complexity and the variation in epidermal chemical composition, specifically, the CHCs. With regard to their distribution characteristics, CHCs are located in the outermost layer of the insect cuticle [39]. They are the first interfacial substances to interact with droplets when they come into contact with the body surface. Due to their strong hydrophobicity, the repulsion of droplets by CHCs precedes the physical structure of the cuticle itself [6]. The experimental results obtained demonstrated that lipid and cationic adjuvant treatments significantly altered the characteristic signals of CHCs in the epidermis of larvae of different instars, but had little effect on protein- and chitin-related signals. This outcome validates the wetting mechanism mentioned above, which indicates that the interactions, including dissolution and chemical reaction, between CHCs and these two classes of adjuvants are instrumental in facilitating their penetration within the epidermis. Furthermore, mid-infrared spectroscopic analyses revealed significant differences in the composition of CHCs in larvae of different developmental stages, which would theoretically lead to variation in the wetting effect. It is noteworthy that CHCs not only function as hydrophobic barriers but also play a pivotal role in chemical communication (e.g., interspecific recognition, sex signaling, social insect rank differentiation) and pathogenic microbial defense [40,41]. Consequently, the dynamics of CHCs during development may act as a regulatory node for organisms to coordinate hydrophobic adaptation with other physiological functions [42]. This phenomenon mirrors the multifunctionality of the chitin microstructure [30], and together they reveal an integrative strategy in the ecological adaptation of organismal surface functions.

The functional morphology features of the epidermis of *P. polytes* larvae documented in this study can provide fundamental information for biomimetic materials and robots with different wettability requirements. The high wettability of silicone adjuvants in the epidermis of *P. polytes* larvae of different instars, as found, suggests the potential of silicone-based adjuvants for the application of pesticide delivery systems in modern agriculture. In addition, the dominant role of structure in epidermal wettability, demonstrated in this study, suggests that the deposition efficiency of pesticides at complex biological interfaces can be optimized by designing biomimetic adjuvants that mimic the topological features of the natural epidermis. Further studies incorporating the dynamic spreading process of adjuvant molecules on the nanolattice to model wetting, in order to resolve the screening mechanism of biological microstructure for multiple classes of adjuvants, will also provide a new paradigm for the design of dynamic wettability materials.

## Figures and Tables

**Table 1:** The contact angle of liquids on epidermal surface of 4th and 5th instar larvae of *P. polytes* (Mean ± SE).

Liquid Type	Liquid Name	4th Instar	5th Instar
Water	Water	98.03 ± 3.37° Ba	126.94 ± 6.47° Aa
Lipidic Adjuvants	1.0% methylated seed oil	48.44 ± 3.90° Acd	32.99 ± 7.94° Bd
	0.1% octodecyl glucoside	53.14 ± 5.77° Ac	21.92 ± 3.59° Be
	97% mineral oil	65.40 ± 4.98° Ab	44.65 ± 10.73° Ac
Organosilicon Adjuvants	0.03% Silwet 618	48.04 ± 5.77° Acd	30.82 ± 7.68° Ac
	0.03% Silwet 408	44.55 ± 5.63° Ade	15.50 ± 4.53° Be
Cationic Adjuvants	0.9 g/L benzalkonium chloride	42.16 ± 6.29° Ae	60.75 ± 12.40° Ab
	1.0 g/L tetradecyl benzyl dimethyl ammonium chloride	33.06 ± 4.67° Bf	44.65 ± 6.27° Ac

Notes. Same capital letters indicate no significant differences in the target liquid between the two instar larvae (*p* > 0.05). Same lower-case letters indicate no significant differences in the target instar larvae between different liquids (*p* > 0.05).

## Data Availability

The data presented in this study has been uploaded to Mendeley Data and are now openly available at: 10.17632/53wcyw3r64.1.

## Supplementary Materials

The following supporting information can be downloaded at: https://www.mdpi.com/article/10.3390/biomimetics10040251/s1, Movies of the simulations of water, 1% methylated seed oil (representative of lipids), 0.9 g/L benzalkonium chloride (representative of cationic adjuvants) and 0.03% Silwet 618 (representative of organosilicon adjuvants) dropping and bouncing on the epidermis of 5th in-star larvae of *P. polytes*, with a time span of 500 ms. The color indicates the pressure dis-tribution of the droplets, the closer the color is to red, the higher the pressure, the closer the color is to blue, the lower the pressure. Movie A1: Simulations of water. Movie A2: 1% methylated seed oil. Movie A3: 0.9 g/L benzalkonium chloride. Movie A4: 0.03% Silwet 618.

## Abbreviations

The following abbreviations are used in this manuscript:

CAH	Contact Angle Hysteresis
CHCs	Cuticle hydrocarbons
SEM	Scanning Electron Microscope
20E	Ecdysone
JH	Juvenile hormone
CHS	Chalcone synthase gene
CPR	Cytochrome P450 reductase gene

## Appendix A

**Table A1:** Structural characteristics of the protuberances on the epidermis of 3rd and 4th instar larvae of *P. polytes* after soaking (Mean ± SE).

Instar	Treatments	Base width (μm)	Height (μm)	Chamfer Angle (°)	Spacing (μm)
3rd	Hexyl hydride	0.73 ± 0.04	0.66 ± 0.03	38.65 ± 0.50	1.25 ± 0.18
	1.0% methylated seed oil	0.84 ± 0.05	0.62 ± 0.09	41.06 ± 9.73	1.16 ± 0.23
	0.9 g/L benzalkonium chloride	0.74 ± 0.09	0.66 ± 0.14	37.17 ± 0.43	1.35 ± 0.13
	0.03% Silwet 618	0.67 ± 0.09	0.58 ± 0.04	36.98 ± 9.52	1.13 ± 0.11
4th	Hexyl hydride	0.91 ± 0.03	0.70 ± 0.01	39.97 ± 0.57	1.29 ± 0.03
	1.0% methylated seed oil	0.92 ± 0.10	0.69 ± 0.24	39.61 ± 11.19	1.14 ± 0.08
	0.9 g/L benzalkonium chloride	0.90 ± 0.34	0.71 ± 0.16	39.28 ± 4.69	1.38 ± 0.06
	0.03% Silwet 618	0.92 ± 0.10	0.61 ± 0.08	40.53 ± 2.19	1.16 ± 0.14

**Table A2:** Structural characteristics of the protuberances on the epidermis of 5th instar larvae of *P. polytes* after soaking (Mean ± SE).

Treatments	Base Width (μm)	Tip Width (μm)	Base Height (μm)	Lobe Height (μm)	Angle Between Lobes (3-Lobed)	Angle Between Lobes (2-Lobed)
Hexyl hydride	1.16 ± 0.01	1.19 ± 0.02	1.80 ± 0.08	0.50 ± 0.02	49.51 ± 0.52	60.17 ± 0.31
1.0% methylated seed oil	1.17 ± 0.26	1.15 ± 0.42	1.69 ± 0.18	0.49 ± 0.01	48.35 ± 0.69	59.90 ± 1.84
0.9 g/L benzalkonium chloride	1.16 ± 0.36	1.22 ± 0.02	1.71 ± 0.26	0.47 ± 0.25	49.07 ± 1.91	60.49 ± 0.28
0.03% Silwet 618	1.14 ± 0.16	1.18 ± 0.37	1.90 ± 0.37	0.55 ± 0.28	49.57 ± 1.20	60.62 ± 3.08
